# The Role of Bacteria-Derived Hydrogen Sulfide in Multiple Axes of Disease

**DOI:** 10.3390/ijms26073340

**Published:** 2025-04-03

**Authors:** Aleksandr Birg, Henry C. Lin

**Affiliations:** 1 Medicine Service, New Mexico VA Health Care System, Albuquerque, NM 87108, USA; helin@salud.unm.edu; 2 Division of Gastroenterology and Hepatology, University of New Mexico, Albuquerque, NM 87106, USA

**Keywords:** brain–gut axis, microbiome, hydrogen sulfide, dysbiosis, gut–immune axis, gut–heart axis, gut- endocrine axis

## Abstract

In this review article, we discuss and explore the role of bacteria-derived hydrogen sulfide. Hydrogen sulfide is a signaling molecule produced endogenously that plays an important role in health and disease. It is also produced by the gut microbiome. In the setting of microbial disturbances leading to disruption of intestinal homeostasis (dysbiosis), the concentration of available hydrogen sulfide can also vary leading to pathologic sequelae. The brain–gut axis is the original studied paradigm of gut microbiome and host interaction. In recent years, our understanding of microbial and host interaction has expanded greatly to include specific pathways that have branched into their own axes. These axes share a principal concept of microbiota changes, intestinal permeability, and an inflammatory response, some of which are modulated by hydrogen sulfide (H_2_S). In this review, we will discuss multiple axes including the gut–immune, gut–heart, and gut–endocrine axes. We will evaluate the role of H_2_S in modulation of intestinal barrier, mucosal healing in intestinal inflammation and tumor genesis. We will also explore the role of H_2_S in alpha-synuclein aggregation and ischemic injury. Finally, we will discuss H_2_S in the setting of metabolic syndrome as int pertains to hypertension, atherosclerosis and glucose-like peptide-1 activity. Majority of studies that evaluate hydrogen sulfide focus on endogenous production; the role of this review is to examine the lesser-known bacteria-derived source of hydrogen sulfide in the progression of diseases as it relates to these axes.

## 1. Introduction

The gut microbiome contributes significantly to health and drives disease. Hydrogen sulfide, a gaseous neurotransmitter, is a key mediator of these effects. H_2_S is generated both endogenously by mammalian cells and exogenously by gut bacteria. While much is known about the role of endogenous H_2_S in maintaining physiological homeostasis, the role of bacteria-derived H_2_S in human disease is less well understood. Bacteria-derived H_2_S is produced by dissimilatory reductase (dsr) of sulfate-reducing bacteria (SRB) and by cysteine desulfhydrase of *Fusobacteria*. Excessive exposure to bacteria-derived H_2_S occurs in the setting of a perturbed gut microbiome, known as gut dysbiosis [1]. In this review, we will explore the role of bacteria-derived H_2_S in human disease.

An archetype of communication between the gut microbiome and the host is the brain–gut axis (BGA) [2,3]. The BGA is involved in health and disease. Recent studies have shown the role of the gut microbiome in progression of diseases involving the brain, including Parkinson’s Disease, Alzheimer’s Disease, and schizophrenia [2]. The gut microbiome, through signaling molecules such as H_2_S, can communicate directly or indirectly with the central nervous system [4]. H_2_S entering the circulation could act directly on the brain. H_2_S in the gut could also act indirectly by using the enteric nervous system and the extrinsic nerves, such as the vagus nerve, for signal transmission to the brain [2,4].

**Figure 1:**
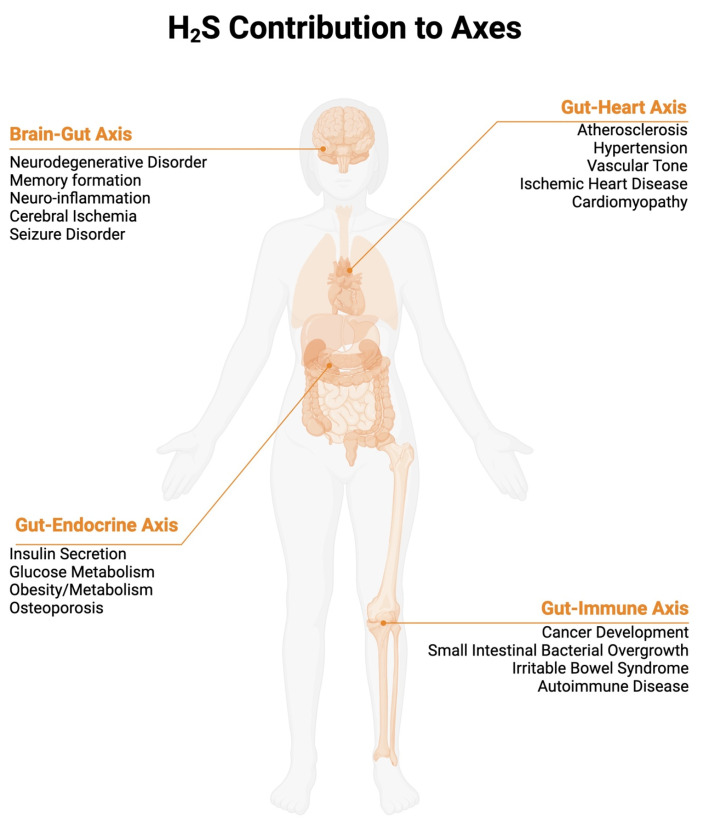
H_2_S contribution to Axes. While not comprehensive, the figure shows 4 axes discussed in this review where most information is available regarding the bacteria-derived role of H_2_S. Below each axis, associated diseases states discussed in this review are displayed. Created in BioRender (https://www.biorender.com/, accessed on 1 December 2024).

The reach of the gut microbiome to different organ systems is seemingly without limit and has been described as the “Gut–immune Axis”, “Brain–Gut Axis”, “Gut–heart Axis”, and “Gut–endocrine Axis” [5,6] (Figure 1).

### 1.1. Endogenous H_2_S

Endogenous hydrogen sulfide is a signaling molecule produced by eukaryotic cells with an extensive role in regulating physiological functions, including the modulation of vasodilation, memory, angiogenesis, anticoagulation [7], immune response [8], insulin secretion, motility, bone metabolism, oxygen sensing, neurotransmission, erection, and pain sensation [9]. H_2_S is produced in multiple cell types including cardiovascular endothelial cells, hepatocytes, pancreatic beta cells, enterocytes, and neurons via four enzymes: cystathionine β-synthase (CBS), cystathionine γ-lyase (CTH or CSE), 3-mercaptopyruvate sulfurtransferase (3MST), and Selenium-Binding Protein 1 (SELENPB1) [10,11,12]. CBS and CSE are involved in the reaction whereby the sulfur-containing bond in L-cysteine is cleaved leading to the production of hydrogen sulfide as a byproduct. CBS and CSE also convert homocysteine to cysteine via a transsulfuration pathway in a pyridoxal-5 phosphate dependent manner [10]. Initial H_2_S studies focused on endogenous role in the vascular system and vascular smooth muscle cell modulation [7], which has provided more data in this area compared to other systems.

One frequently debated topic is physiologic plasma H_2_S concentration, as early methods using methylene blue were often inaccurate and insensitive [13]. These methods often overestimate the concentrations present. More novel methods to measure H_2_S are more accurate and can differentiate between different pools of H_2_S [14]. It is also possible that these early studies overestimated the concentration of endogenous H_2_S due to the contribution of gut-bacteria-derived H_2_S that increased the overall measured concentration of this gas. The accurate measurement of the production of endogenous or gut bacteria-derived H_2_S remains inconsistent and the role of bacteria-derived H_2_S is often not well addressed in the current literature.

### 1.2. Bacteria-Derived H_2_S

The intestinal microbiome is an important source of H_2_S. Recent reviews have been published on the role of H_2_S-producing bacteria in the regulation of health [15]; this limited scope has not evaluated the function of hydrogen sulfide as the stimulus. Bacteria-derived H_2_S can permeate across the intestinal epithelium [16]. Hydrogen sulfide is also a product of sulfate-reducing bacteria (SRB) produced as a final metabolite of dissimilatory sulfate reduction (DSR) process [17]. SRB are habitants of anoxic environment in the intestinal lumen reducing sulfate to hydrogen sulfate; SRB are primarily Gram-negative with few Gram-positive species [18]. SRB utilize hydrogen for sulfate reduction using lactate, pyruvate, malate, succinate and acetate as electron donors [19]. Sulfate is the terminal electron acceptor in the DSR pathway [20]. SRB are grouped together based on their metabolic function; the list of genus in the SRB group is constantly growing and includes Archaeoglobus, Deltaproteobacteria, Thermodesulfovibrio, Desulfotomaculum [20]; *Desulfovibrio* species remains one of the most studied and well characterized in the SRB group [21,22]. H_2_S can be toxic (even to SRB at high enough concentrations), the DSR pathway can withstand concentrations up to 25 mM prior to H_2_S becoming bactericidal. In addition to SRB, hydrogen sulfide is generated by *Fusobacteria* such as *Fusobacterium nucleatum* from amino acids such as L-cysteine and peptides such as glutathione. This reaction, whereby cysteine is converted to H_2_S, pyruvate, and ammonia, depends on the enzyme L-cystine desulfhydrase and the coenzyme pyridoxal-5‘phosphate [23,24]; these reactions are dependent on B6 bioavailability, which plays an important role in sulfurous amino acid metabolism [25].

### 1.3. Detoxification of H_2_S

**Figure 2:**
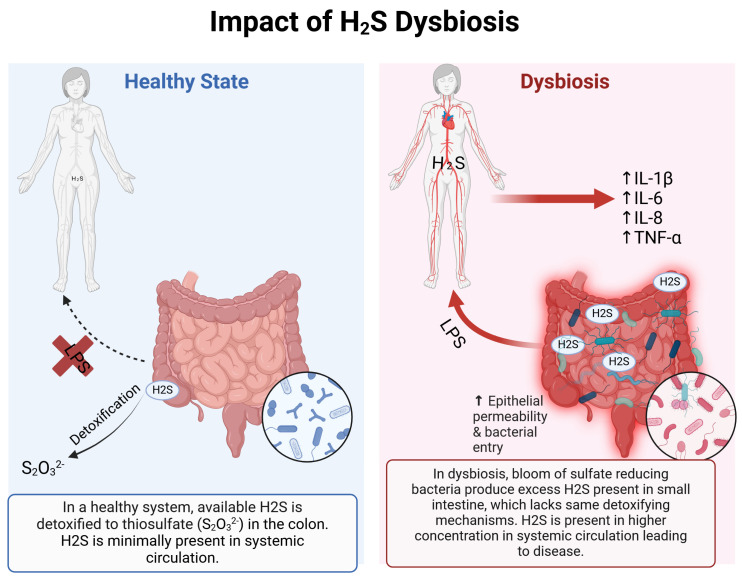
Impact of H_2_S Dysbiosis. (**Left**) Healthy states showing minimal H_2_S plasma concentration and colonic concentration. Mechanisms showing H_2_S detoxification to avoid intestinal barrier breakdown and microbial metabolite permeability (represented at LPS, lipopolysaccharide). (**Right**) Dysbiosis state increased H_2_S concentration in gastrointestinal tract, leading to epithelial barrier breakdown and microbial transition into systemic circulation. Increase H_2_S plasma concentration is expected to lead to inflammatory response. Created in BioRender.

Normally, the gut microbiome is mostly confined to the colon and the distal small intestine, with concentrations falling from 10^12^/mL in the colon to 10^2^/mL or less in the jejunum and duodenum [26]. As result of this compartmentalized distribution, most of the gas production and metabolism occurs in the large intestine where H_2_S concentrations can reach 1000 parts per million (ppm) [27] or 1–2.4 mM in a healthy state [17]. The colonic concentration of H_2_S is higher than the lethal concentration of H_2_S, reported at 800 ppm when exposed for 5 min [28]. Such large amounts of H_2_S can be handled by the colon due to its significant detoxifying capacity that converts H_2_S to thiosulfate, a nontoxic metabolite; colonocytes utilize H_2_S as an energy source in the respiratory chain production of ATP. Similar detoxification system in the liver and kidneys also oxidize H_2_S to thiosulfate and other sulfated molecules [28]. Under healthy conditions, publications report a baseline free plasma H_2_S concentrations around 370 nmol/L [29]. The detoxification of bacteria-derived H_2_S becomes challenged in the setting of gut dysbiosis as represented by small intestinal bacterial overgrowth (SIBO) where the compartmentalization of the gut microbiome is lost leading to its expansion into the more proximal regions of the gastrointestinal tract (Figure 2). SIBO has been well described in several GI conditions as the potential driving mechanism for disease [30]. In the setting of the expansion of the microbiota into the small intestine, the handling of H_2_S becomes much more challenging as the detoxifying properties of small intestine are reported to be 1/20th compared to the colon [31] resulting in the entry of more bacteria-derived H_2_S into the circulation from the gut. At high enough concentrations, H_2_S may inhibit the cytochrome C oxidase and impair ATP production [32]. H_2_S in the small intestine can lead to downstream sequelae, not seen when H_2_S is confined to the colon, due to the limited ability of small intestinal mechanisms to detoxify [28]. The presence of bacteria in the small intestine triggers defensive hyperperistalsis and hypersecretion to squeeze and flush out the microbes accounting for the complaint of diarrhea and rumbling abdominal sounds by patients with SIBO.

### 1.4. H_2_S and Disease

Over the last few decades, the importance of hydrogen sulfide has come into view as a signaling molecule effecting many organ systems [33]. While the influence of endogenous H_2_S dominates our understanding of the role of this gas, the impact of bacteria- derived H_2_S is poorly understood. Some studies have evaluated the role of bacteria-derived H_2_S by exploring the effects of administering into the gut a donor molecule that releases H_2_S. Experimentally administeredH_2_S-donating molecules do not always represent the gut luminal environment of bacteria-derived H_2_S nor its release and pattern of removal.

Concentrations of H_2_S in the colon, recorded as high as 250 M, often reaching 40 M in the cecum, derived from the gut microbiome can produce a negative impact on the surrounding tissue [16,34]. Exposure to excessively high concentrations of H_2_S has been shown to inhibit mitochondrial respiration, reduce intracellular redox environment, and inhibit cellular functions such as maintaining the integrity of intestinal mucus via a reduction in disulfide bonds [35,36].

The effects of H_2_S may be both good and bad, depending on the concentration of this gas. In contrast to the toxic effects, such as inhibition of respiration and low concentrations of H_2_S, whether endogenous or exogenous, can be beneficial and has been shown to stabilize the mucus biofilm layer, prevent bacterial adherence to the biofilm, and prevent invasion of pathogens through the epithelial layer [34]. With H_2_S varying by source and concentration, it remains a challenge to predict the effects of H_2_S in the gut environment. It is possible that endogenous and gut-bacteria-derived H_2_S play a symbiotic, protective role until an event leading to disruption of the gut microbiome such as SIBO resulting in the disruption of homeostasis leading to overgrowth of sulfate-reducing bacteria triggering disease [37].

Using next generation DNA sequencing techniques targeting bacterial 16S rRNA genes, a large portion of previously uncultured gut microbiome has been explored and identified, allowing for an improved understanding of metagenomics and metabolomics [38]. With this advance, it has become clear that a bloom of hydrogen sulfide-producing bacteria is a common feature of a perturbed gut microbiome; however, the impact of an overgrowth of these bacteria and the effect of exposure to excessive amounts of bacteria-derived H_2_S are not known.

## 2. Effects of Hydrogen Sulfide in Different Axes

### 2.1. Gut–Immune Axis/Inflammatory Bowel Disease

Dysbiosis leads to increased intestinal permeability allowing for microbial translocation and activation of systemic inflammation [39,40]. Dysbiosis in animal models leads to a decrease in tight junction protein occludin and an increase in Claudin-2, a pattern of changes characteristic of leaky gut that can lead to endotoxemia [41]. Increased intestinal permeability (leaky gut) leads to entry of endotoxins such as lipopolysaccharide (LPS) into the portal then systemic blood [19] triggering a proinflammatory response from the immune system [42]. Endotoxin activates toll-like receptor 4-mediated signaling, the release of proinflammatory cytokine, and increased oxidative stress. Endotoxemia with systemic inflammation is linked to glucose intolerance, hyperlipidemia and hypertension of metabolic syndrome. By presenting microbes and microbial antigens to immune cells, such as hepatic Kupffer cells, translocation across the intestinal barrier upregulates proinflammatory cytokines, such as interleuken-6 (IL-6) and tumor necrosis factor a (TNF-a) via TLR4 pathway.

Gut epithelium serves as a physical barrier against pathogens and harmful metabolites in the gut lumen [16]. Both endogenous and bacteria-derived H_2_S can modulate microbial translocation by impacting the intestinal barrier.

H_2_S has been reported to support the intestinal barrier, enhance mucosal defense against pathogens, promote the healing of mucosal ulceration, and facilitate the resolution of intestinal inflammation [9,43,44]. The gut epithelium in contact with the microbiome can both produce and remove H_2_S. The small amounts of endogenous H_2_S produced by the intestinal cells is dwarfed by the massive amounts of bacteria-derived H_2_S produced by sulfate-reducing bacteria and *Fusobacteria*. Intestinal epithelial cells also remove H_2_S through its detoxification system so that luminal H_2_S is used to generate ATP as a metabolic barrier to any H_2_S that passively diffuse through the intestinal wall after its passage through the biofilm layer [42]. The downstream effects of the bacteria-derived H_2_S on other organ systems (see later section) depends on the combined effects of downregulation of mitochondrial proteins responsible for H_2_S detoxification [45] and exposure to excessive amounts of H_2_S excess in dysbiosis [46]. Downregulation of detoxifying protein has been shown in pediatric patients with Crohn’s disease leading to the depletion of butyrate-producing-bacteria, which leads to overgrowth of H_2_S-producing bacteria [45].

In the setting of small intestinal bacterial overgrowth, the more proximal regions of the gastrointestinal tract are exposed to high concentrations of H_2_S. In a study by Parajuli et al., looking at the effects of a donor of exogenous H_2_S on; high concentrations of H_2_S (500 µM to 1 mM) lead to inhibition of pacemaker activity of interstitial cells of Cajal in a mouse model with suppression of amplitude and frequency [47]. Exposure to a high but not cytotoxic concentration of H_2_S to the small intestinal human cell line led to increased inflammatory response with increased IL-8 expression and DNA damage by generating excessive oxidative species [48]. A more recent study by Kushlevych et al. evaluating bacteria-derived H_2_S generated by SRB showed that an increase SRB and by extension, exposure to higher concentration of luminal H_2_S, can lead to ulcerative colitis-like changes such as H_2_S-inhibited colonocyte growth, increased phagocytosis, increased intestinal bacteria death, and induced hyperproliferation of intestinal epithelial cells [35].

The effects of H_2_S in the colon appear to be protective rather than harmful. In a study by Motta et al., administration of exogenous H_2_S in the setting of colitis led to restoration of the microbial biofilm and increased production of mucus granules [49]. Increased H_2_S concentrations were also shown to reduce neutrophil infiltration and maintain the thickness of the mucus layer [49]. Exogenous H_2_S administration in an enterocolitis model showed improved intestinal perfusion and reduced bowel injury [50]. Colonocytes may respond differently to H_2_S as these cells are able to utilize H_2_S as an energy source for generating ATP in the setting of hypoxia and are able to withstand concentrations of H_2_S up to 50 µM [42,51].

Interestingly, the effects of H_2_S-producing bacteria have been shown to have the opposite effect to the gas. H_2_S-producing bacteria appear to be harmful to the colon as increased upregulation of Th17 and Treg cells with increased cytokine production is seen in germ-free mice colonized with *Desulfovibrio indonesiensis* [1]. This proinflammatory effect was exacerbated by the administration of the SRB mixture collected from patients with colitis. Similarly, Figliuolo et al. showed that the administration of *Desulfovibrio indonesiensis* further significantly exacerbated the injury to the colonic architecture [1]. The differences showcased here in the effects of H_2_S-producing bacteria compared to H_2_S gas remain unclear.

Gut dysbiosis is a key contributor to activation of intestinal immune system driving the pathogenesis of conditions associated with intestinal epithelial inflammation, including inflammatory bowel disease [38]. Concentration of bacteria-derived H_2_S is elevated in patients with ulcerative colitis and Crohn’s Disease [52] although it remains unclear if this is a cause or effect as part of intestinal inflammation. It has been thought to be particularly prevalent in IBD as SRB generally cannot survive in an acidic environment [17]. However, in the setting of IBD, the luminal pH of the colon is higher than that of healthy controls [53] higher pH allowing for a more favorable growth environment for SRB. Intestinal bacteria, and specifically SRB, utilize short chain fatty acids, such as butyrate, for fermentation and sulfate reduction [17]. Short chain fatty acids are produced by the gut microbiome by fermentation plays a significant role in maintaining the physiology of normal, healthy mucosa [20,54]. These fatty acids are an important energy source to the epithelium and its production is impaired in inflammatory bowel disease associated with intestinal injury [54,55]. H_2_S can lead to intestinal mucosal damage via oxidation of intestinal butyrate in the mitochondria, leading to a starvation effect [52]. Not only do increased H_2_S levels lead to the oxidation of available butyrate, but elevated hydrogen sulfide is generally considered toxic to bacteria [17], which can lead to dysbiosis.

- Colorectal Cancer development

Multiple H_2_S-generating bacteria have been implicated in development of cancer (i.e., breast, colon) [56] via production of hydrogen sulfide. In setting of oncogenesis in colorectal cancer, H_2_S has multiple functions known to promote tumor growth including regulation of vascular function and angiogenesis [7], regulation of electron transport and cellular metabolism [57,58], regulation of intracellular signaling and apoptosis [59,60]. Exposure to exogenous H_2_S at concentrations of 50–200 µM can lead to accelerated cell cycle progression by increasing the S-phase of cells and decreasing levels of p21 [61]. However, much higher H_2_S concentrations have been shown to suppress cells growth by upregulating p21 expression [61].

H_2_S is known to be genotoxic, which can lead to chromosomal instability at concentrations in the regions of 250 µM in the colon [48,61,62]. This concentration is commonly reported in healthy colons [17,34,48] without a dysbiotic state; therefore, dysbiosis in the intestines can lead to much higher concentrations of H_2_S exposure [1] leading further drive toward oncogenesis. When combined with other mutations that impact DNA repair, high concentrations of H_2_S can be carcinogenic via the Ras/MAPK pathway, leading to interference in mitochondrial function. The Ras/MAPK pathways is a well-described mechanism of carcinogenesis in many cancers [36]. Similar reports are described in melanoma progression with exogenous H_2_S leading to the inhibition of MAPK pathways, common process in melanoma cells [63]. Since most of the literature is based on studies utilizing exogenous sources of H_2_S to test the effect of gas on cancer progression, the role of bacteria-derived H_2_S is not well established. The presence of a high density of H_2_S-generating *Fusobacterium nucleatum* has been shown in colorectal cancer [64]. Poor prognosis, metastatic disease, and recurrence are also linked to high concentrations of *Fusobacterium nucleatum* in colorectal tumor tissues [65]. Interestingly, out of the two known clades of *Fusobacterium nucleatum* (*Fn*), only one clade within the subspecies of animalis (Fna C2) drives colorectal tumorigenesis [66].

- Irritable Bowel Syndrome/SIBO

SIBO is recognized as excessive bacteria in the small intestine [67], although more recent studies have also noted that bacterial growth is not limited to the small bowel and can be seen as overgrowth in the colon [68]. Prior studies in SIBO explored the role of the gut microbiome and its gas metabolites produced: hydrogen and methane [69] as major modulators of gut motility and transit [68,70]. Hydrogen and methane gases are used as an indirect diagnosis of SIBO using lactulose breath testing by testing for the presence of abnormal profiles of bacteria-derived gases in the exhaled breath. Breath H_2_S has been shown to improve the accuracy of clinical interpretation of breath testing as ~60% of the population depends on sulfate-reducing bacteria for consumption of hydrogen generated from bacterial fermentation [71]. Prior to the identification of the role of gut bacteria in IBS [72], Irritable Bowel Syndrome (IBS) was considered a functional GI condition identified by clinical criteria tied to with altered intestinal motility and abdominal pain. While the pathogenesis of IBS is still controversial, there is an important role for gut dysbiosis as characterized by increased prevalence of abnormal breath test results in IBS [73]. Many patients with IBS report worsening of their symptoms during times of stress. This association can be understood based on the induction of growth of sulfate-reducing bacteria by the stress amine norepinephrine [74] and its reversal by magnesium oxide [75].

Exposure to excessive bacteria-derived H_2_S has been proposed to cause intestinal epithelial injury and breakdown of the mucus barrier [1,76] as SRB reduce disulfide bonds which can denature the protective mucin in the biofilm [77] that normally lowers the probability of bacterial translocation by separating the epithelial layer from the luminal microbiota [78,79]. Animal studies involving the colonization of SRB showed increased cellular inflammation of the mucosal layer as well as upregulated expression of inflammatory cytokines [1,80,81,82]. While SRB-derived H_2_S could produce epithelial inflammation and breakdown at very high concentrations; this level of injury is not present in dysbiosis and not required for the induction of leaky gut. Instead, a novel H_2_S-independent mechanism driven by SRB has recently been described that induces increased intestinal permeability via the Snail transcription factor [83] and increase immune activation [84]. This mechanism may explain the leaky gut and microbial translocation associated with SIBO where there is no evidence of overt epithelial disruption or severe mucosal inflammation.

The role of gut-bacteria-derived hydrogen sulfide remains poorly understood in SIBO/dysbiosis. A recent study by Birg et al. showed that including H_2_S as a measured metabolite provides a more comprehensive look at the gas profile generated by the gut microbiome [85]. A recent case registry by Goldenberg et al. evaluated patients suspected of having SIBO. These authors found that 42% of these patients had diarrhea when their breath test was positive for H_2_S [86]. Singer et al. showed similar results with increased breath H_2_S concentration on lactulose breath testing correlating with diarrhea [87].

Tissue samples collected from patients with IBS showed evidence of inflammatory response [88,89]. Similar immune activation is seen in patients with SIBO as they have an elevated combination of IL-1B, IL-6, and TNF-a concentrations in the duodenum [90]. IBS is considered, in general, to have ‘controlled’ inflammation as seen in normal mucosa without pathologic inflammatory response as can be seen with inflammatory bowel disease or infectious pathogen. The region of the gut investigated is a factor in variable reports of immune activation in patients with IBS as the small intestine rather than the colon is likely to be the critical site [91]. The presence or absence of bacteria-derived hydrogen sulfide may also be a factor that determines the immune response detected in different studies.

- Ulcer healing

Endogenous H_2_S has long been known to protect the gastric mucosa from the cytotoxicity of non-steroidal anti-inflammatory drug (NSAID) [44] via inhibition of leukocyte adherence to epithelium [44]. In a study by Wallace et al., rats treated with proton-pump inhibitor (PPI) showed evidence of increased NSAID-induced mucosal injury [92] when production of H_2_S was doubled even as the enzymes for endogenous H_2_S synthesis (cystathione gamma lyase (CSE) and cystathione beta synthase (CBS) was not changed [92]. The higher amount of H_2_S must have come from gut bacteria as PPI therapy increases SRB proliferation in otherwise healthy subjects [93]. Since PPI therapy is associated with the development of SIBO [94], increased exposure to bacteria-derived H_2_S may account with the increased NSAID-induced mucosal injury when treated with these acid suppressive agents. While these studies suggest a role for bacteria derived- H_2_S and mucosal injury, more research is needed to show cause and effect.

### 2.2. Brain–Gut Axis

In a healthy state, H_2_S plays a significant and beneficial role in neurologic function. This gaseous neurotransmitter is responsible for the long-term potentiation of hippocampal neurons [95], an experimental outcome that correlates with the laying down of memory. It is also a cytoprotectant [96]. Hydrogen sulfide can freely cross the blood–brain barrier and act as a neuromodulator by enhancing NMDA receptors [8,97]. H_2_S can also directly modulate neurons by modifying intracellular pH and calcium levels [8,98]. H_2_S protects the blood–brain barrier by suppressing local reactive oxygen species formation and local inflammation [99].

The brain–gut axis (BGA) refers to a bidirectional connection between the GI tract and the central nervous system [100]. This communication occurs via signaling molecules across multiple pathways. The brain to gut signaling mediate motor, sensory and secretory functions of the intestinal tract [2]; the gut to brain axis impacts cognitive and neurobehavioral functions [101]. BGA plays an important role in neuroinflammation that can be modulated by gut-luminal H_2_S. In addition to the role of H_2_S in epithelial cell integrity and mucosal barrier disruption (as discussed above), intestinal H_2_S also plays a role in the enteric nervous system. H_2_S acts on the vanilloid-1 receptors on afferent terminals of the GI tract [8]. H_2_S also causes excitation potentials of the sensory neurons in an animal model with colitis, deemed to be a protective property in animals [102] (not yet evaluated in clinical studies). Hydrogen sulfide clearly plays a significant role in the bidirectional communication of BGA; however, the data on gut-derived H_2_S in the nervous system remains poorly studied. The immune system often acts as a mediator and communication pathway between the microbiome and central nervous system. Endotoxemia as a consequence of translocation of gut microbes of microbial products is associated with systemic inflammation and neuroinflammation [41]. Germ free mouse models have shown that increased permeability in the blood–brain barrier is seen in germ free animals compared to controls via reduction in tight junction proteins [103]. Germ free animal models have also been shown to have an increased number of immature microglia in the central nervous system [104], with alterations to the microglia function that has been linked to neurodegenerative disorders [105,106].

- Parkinson’s Disease

The role of the gut microbiome in Parkinson’s Disease (PD) has been recognized for years; more recently, an increased number of sulfate-reducing bacteria has been found in the stool of patients with PD. The H_2_S concentrations in cerebrospinal fluid were higher in patients with PD compared to healthy controls [107]. While this finding alone does not directly prove that cerebrospinal fluid H_2_S is derived from the gut-microbiome, patients with PD have been found to have altered microbiome with increased potential for H_2_S secretion with overgrowth of *A. muciniphila* and *B. wadsworthia* species [108]. In the setting of SIBO where gut-luminal H_2_S concentrations can be increased, leading to H_2_S excess; H_2_S can passively diffuse into the systemic circulation, eventually able to cross the blood–brain barrier [107]. High concentrations of H_2_S can lead to reactive oxygen species production, which can lead to the development of alpha-synuclein oligomers aggregation in neurons [107]. Findings related to the concentration of endogenous H_2_S may be contrary, as a rat model for PD showed decreased levels of H_2_S in the substantia nigra and striatum. Administering exogenous H_2_S donors in this animal model led to slowing of the progression of the motor changes in a movement disorder. Additionally, the addition of H_2_S donor leads inhibition of accumulation of proinflammatory cytokines like TNF-α in the substantia nigra [109].

The connection between SRB and H_2_S blood concentration is observational at best, as no study has reported blood H_2_S concentrations in the setting of neuroinflammation and dysbiosis. Previous studies have shown a decrease in CD8+ T-lymphocytes in PD patients, which may be driven by high H_2_S concentrations, which have shown to induce cell death of peripheral lymphocytes, specifically targeting CD8+ T lymphocytes and natural killer cells [107]. Multiple studies have reported increases in H_2_S-producing genera (*Prevotella*, *Porphyromonas*), while butyrate producing genera (*Roseburia*, *Blautia*, *Faecalibacterium*, *Moryella*, *Faecalibacterium*, *Anaerostipes*) are decreased [110,111]. Of particular importance in the colon is several SRB falling in the *Desulfovibrio* genus, as this genera has been reported in multiple PD studies in overabundance [107]. Since migration of gut resident bacteria to the brain via the vagus has been shown to cause microglial activation in mice [112], resident gut bacteria, such as those that generate H_2_S, could conceivably reach the brain to directly trigger neuroinflammation and be responsible for degenerative brain diseases.

While no studies directly evaluated the role of dysbiosis in progression and advancement of GWS cognitive symptoms, correlations can be seen in Parkinson’s disease. PD has been shown to have increased latencies in cerebrovascular reactivity (cerebrovascular blood flow) using magnetic resonance imaging [113]. Cerebrovascular reactivity changes have similarly been reported in similar patient populations with cognitive deficits (i.e., Gulf War Syndrome, traumatic brain injury) [114]. Interestingly, PD patients were found to have increased permeability to LPS and intestinal permeability as can be seen in dysbiosis [115,116]. Dysbiosis has been reported to contribute to neuro and systemic inflammation and increasing both the intestinal and blood–brain barrier permeability. Bacterial products, such as LPS, can increase neuroinflammation by increasing barrier permeability [117,118]. Interestingly, recent works have shown that neuroinflammation via microglial cells modulates sodium retention and systemic blood pressure, leading to abnormal hypertensive response in a dysbiosis state [119].

- Alzheimer’s disease

Alzheimer’s disease (AD) is thought to involve amyloid and tau proteins as drivers of the disease. Current evidence points to the disruption of endogenous H_2_S production in the neurologic tissue [120]. Indeed, in vivo and in vitro study show that H_2_S scavenges the cytotoxic 4-hydroxynonenal product which is increased in AD patients [121]. H_2_S has also been shown to ameliorate amyloid induced damage by reducing the loss of mitochondrial membrane potential and reducing neuroinflammation by inhibiting NF-kB activity [120,122].

While reduced concentration of endogenous H_2_S has been well documented in the role of AD, the impact of gut-derived H_2_S as a source of AD development is not known. Gut dysbiosis has been linked to development of AD and its progression [123]. While dysbiosis associated with AD has been reported to have an increase in H_2_S-generating phylum, *Proteobacteria*, the role of gut-bacteria-derived H_2_S in AD is not well studied [123]. Changes associated with a decrease in short chain fatty acids, such as butyrate, and disruption of intestinal barrier function leading to systemic inflammation has also been described [124].

- Ischemic stroke

Ischemic stroke is caused by a sudden interruption of blood flow to brain tissue [120]. In multiple experimental animal models, the addition of exogenous and large amounts of H_2_S donor lead to a further increase in the infarct volume [125,126] at concentrations of H_2_S significantly above the physiologic concentrations [127]. While this study showed that increased H_2_S levels lead to further hypoxic injury via activation of K_atp_ channels, endotoxemia also increases, with elevated levels of LPS noted due to increased intestinal permeability and intestinal barrier dysfunction in this context [100]. Clinical trials of stroke patients have shown increased abundance of *Desulfovibrio* genus a H_2_S-producing genus [100] but H_2_S production was not measured. However, since it is likely that the increased abundance of SRB in stroke patients will lead to exposure to excessive amounts of H_2_S, there is a high probability that bacteria-derived H_2_S may play a role in worsening the ischemic neurologic damage, but more studies are needed to prove this hypothesis.

### 2.3. Gut–Heart Axis

Cardiovascular diseases are the leading cause of death worldwide. Recent metagenomics analysis identified the gut microbiome as a potential contributor to development of cardiovascular disease. Alterations to the ratio of *Bacteroidetes* to *Firmicutes* and different microbial metabolites, such as short-chain fatty acids, suggest an important role of the gut microbiome in cardiovascular disease progression [128]. One such microbial gas metabolite is hydrogen sulfide. Hydrogen sulfide produces a potent concentration-dependent vasorelaxation via membrane hyperpolarization of vascular smooth muscles [8]. H_2_S can also mediate vasodilation via increasing intracellular cyclic guanosine monophosphate levels and promote the release of nitric oxide leading to vessel dilation [7]. We have previously shown in an animal model that increased exposure to H_2_S in the small but not large intestine uniquely affects the portal circulation leading to portal hyperdynamic blood flow [129]. In vivo studies looking at H_2_S in cerebrovascular flow demonstrated vasodilation, an increase in cerebral blood flow; additionally, studies looking at post stroke changes demonstrated worsening of the post-stroke infarct volume with H_2_S donors [130]. The physiological and pathological impact of exogenous H_2_S may provide a novel target for diagnosing and treating diseases.

- Hypertension

Recent evidence shows the role of gut-derived H_2_S in control of blood pressure; patients with hypertension (systolic pressure > 130 mmHg or diastolic > 80 mmHg) [131] have higher abundance of H_2_S-producing Desulfovibrio [33,132]. Patients with hypertension also have higher abundance of lactate-producing bacteria in the intestinal lumen [8]. SRB utilize lactate as electron donors for sulfate reduction [133]; competition for available lactate can stimulate SRB growth in patients with hypertension [8]. Since exogenous H_2_S can inhibit n-butyrate formation by inhibiting short chain acyl-coA dehydrogenase leading to mucosal barrier disruption [134,135], it is not surprising that patients with hypertension have decreased butyrate and acetate-producing bacteria [8].

Even as a higher number of H_2_S-producing SRB are found in patients with hypertension, direct administration of H_2_S may have a blood pressure-lowering effect, as a study by Hsu et al. showed that pregnant rats gavaged with a H_2_S donor protected the male offspring from hypertension with increased fecal H_2_S concentrations [136]. Interestingly, while levels of SRB are increased in hypertensive states, measured plasma and fecal levels of H_2_S have been reported to be decreased [8]. The measured concentration of H_2_S may depend on the assay as the methylene blue technique may have variable accuracy in measuringH_2_S in plasma [31,137]. Regardless, H_2_S-producing gut bacteria and H_2_S may play a significant part in modulating blood pressure and circulatory system.

- Atherosclerosis

Atherosclerosis is a chronic pathologic cardiovascular disease leading to accumulation of cholesterol-containing macrophage foam cells in arteries [138,139]. Proliferation of vascular smooth muscle cells in arteries is another manifestation of atherosclerosis. H_2_S inhibits vascular smooth muscle cell proliferation which, in turn, inhibits atherosclerosis progression [138]. Information on effects of gut-derived H_2_S on atherosclerosis formation is limited, as most research focused on endogenous produced H_2_S. While bacterial DNA has been found in atherosclerotic plaque formations [140], they are not specific to H_2_S-producing bacteria.

### 2.4. Gut–Endocrine Axis

The gut epithelium consists of many different cell types that are responsible for a multitude of functions including enteroendocrine cells that produce hormones. Even though enteroendocrine cells make up only one percent of intestinal epithelial cells, they play an important role in metabolism and the gut–brain-pancreatic axis [141]. Given their significant role in glucose metabolism, this system has also been called the gut-islet or gut–endocrine axis [142]. Different enteroendocrine cells occupy different regions of the GI tract. Glucose-dependent insulinotropic polypeptide (GIP)-producing K-cells are mainly located in the duodenum, while L-cells producing Glucagon-like peptide-1 (GLP-1) are located in the distal small intestine and colon [143].

- Disturbances in energy metabolism

Digestion and absorption of food occur along the entire length of the small bowel. Products reaching the distal intestinal tract and the normal microbiota include food content that takes more time to assimilate, such as poorly digestible starches, dietary fiber, and bile acids. These luminal contents and short chain fatty acids produced by gut microbiota during fermentation trigger the release of GLP-1 via G_αi/q_ coupled receptor on the apical surface of the L cells [141]. In turn, GLP-1 enhances the release of insulin from pancreatic B-cells in a glucose-dependent stimulation. GLP-1 also regulates B-cell proliferation and inhibits its apoptosis. The gut microbiome is involved in GLP-1 signaling pathway as bile salt hydrolases produced by *Bifidobacterium* can convert conjugated bile salts into deconjugated bile salts to induce further secretion of GLP-1 [142].

This tightly regulated process occurs under a healthy gut microbiome environment to regulate GLP-1 release and insulin production. In contrast, GLP-1 resistance with impaired glucose tolerance was reported in an animal model with gut dysbiosis. Dysbiosis not only decreased GLP-1 production but also reduced the GLP-1 receptor expression [144]. Gut dysbiosis is induced by a high fat diet. It was noted that, when animals were fed such a diet, there was a reduction on GLP-1 receptor expression [145]. The exact mechanism by which dysbiosis led to changes in GLP-1 receptor expression and its metabolic changes were not known [144].

More recent studies have implicated hydrogen sulfide as a possible modulator of GLP-1 activity in the gut; specifically looking at the role of exogenous sources of hydrogen sulfide. There are conflicting reports on the role of hydrogen sulfide. In an animal study by Pichette et al., H_2_S donors administered into the small intestine led to stimulated GLP-1 secretion from L-cells [146]. They also showed that sulfate-reducing bacteria in the gut lumen increased GLP-1 and insulin secretion. In contrast, Qi et al. showed that the introduction of the sulfate-reducing bacteria, *Desulfovibrio*, increased luminal H_2_S levels and inhibited L-cell GLP-1 secretion and gene expression [147]. Reversing the increased H_2_S levels with bismuth subsalicylate improved GLP-1 expression and reversed the inhibitory effect. As the concentration of H_2_S in these two studies was not reported, varying concentrations in H_2_S may explain the opposing results.

Normally, low amounts of endogenous H_2_S are produced by the host. A low level of H_2_S is able to achieve glycemic homeostasis by keeping insulin release at a relatively low level [148] via activation of ATP-sensitive potassium channels in pancreatic β-cells to inhibit insulin secretion. This is achieved by hyperpolarizing the cell membrane [148,149]. Instead of relying solely on more insulin, low doses of endogenous H_2_S is able to maintain good glycemic control by increasing insulin receptor sensitivity and promote glucose uptake by muscle and fat cells [150]. Keeping insulin secretion relatively low helps to protect pancreatic β-cells from apoptosis resulting from their chronic exposure to repeatedly high concentrations of glucose [151]. This tightly regulated system may be disrupted by exposure to excessively large amounts of bacteria-derived H_2_S.

Since administering exogenous H_2_S leads to increased gluconeogenesis, hypertriglyceridemia and fatty liver [152], could exposure to large amounts of H_2_S generated by gut bacteria in dysbiosis lead to insulin insensitivity and hyperglycemia as seen in type 2 diabetes mellitus and metabolic syndrome? Could an SRB seen in dysbiosis be the initiating step? Further studies are needed in this area.

## 3. Conclusions

Hydrogen sulfide is a well-recognized signaling molecule that is tightly regulated to maintain physiological state. H_2_S is also produced by the gut microbiome as a product of several metabolic pathways. While significant focus has been placed on studying the impact of endogenously produced H_2_S over the last three decades, the role of gut-derived H_2_S is less understood. The gut microbiome system can be disrupted, leading to significant changes to the bacterial composition known as dysbiosis. The implication of dysbiosis and changes to H_2_S production and metabolism are not well understood or studied. One of the main limitations in studying hydrogen sulfide in health and disease is being able to differentiate between endogenous and exogenous H_2_S sources. Defining dysbiosis remains controversial to date [153], which creates further limitations in defining an abnormal gut microbiome. Another limitation is not being able to discern between endogenous versus microbial produced H_2_S and the interaction between the two sources on the different axis. A required future study that evaluates the interaction between the two sources will be crucial to our understanding of hydrogen sulfide in health and disease.

In this review, we discuss gut-derived and exogenous H_2_S as a contributor to disease states through different axes. While the brain–gut axis has been the template system for studying the impact of the gut microbiome, there are now findings to suggest the impact of the gut microbiome and its metabolites on many systems. The gut-microbiome-derived hydrogen sulfide is an important producer to total systemic H_2_S concentration that can play a significant role in the different axes. When studying how H_2_S impacts health and disease, it is important for future studies to consider this source to have a comprehensive understanding of the system.

## Data Availability

No new data were created or analyzed in this study.
